# Identification of novel alternative splicing isoform biomarkers and their association with overall survival in colorectal cancer

**DOI:** 10.1186/s12876-020-01288-x

**Published:** 2020-06-05

**Authors:** Haifeng Lian, Aili Wang, Yuanyuan Shen, Qian Wang, Zhenru Zhou, Ranran Zhang, Kun Li, Chengxia Liu, Hongtao Jia

**Affiliations:** 1 Department of Gastroenterology, Binzhou Medical University Hospital (BMUH), No. 662 Huanghe 2nd Road, Binzhou City, Shandong Province People’s Republic of China; 2 Tianjia Genomes Tech CO., LTD., Anhui Chaohu Economic Develop Zone, No. 6 Longquan Road, Hefei, 238014 People’s Republic of China

**Keywords:** Alternative splicing (AS), Colorectal cancer (CRC), Metastasis, RNA-seq, TCGA

## Abstract

**Background:**

Alternative splicing (AS) is an important mechanism of regulating eukaryotic gene expression. Understanding the most common AS events in colorectal cancer (CRC) will help developing diagnostic, prognostic or therapeutic tools in CRC.

**Methods:**

Publicly available RNA-seq data of 28 pairs of CRC and normal tissues and 18 pairs of metastatic and normal tissues were used to identify AS events using PSI and DEXSeq methods.

**Result:**

The highly significant splicing events were used to search a database of The Cancer Genome Atlas (TCGA). We identified AS events in 9 genes in CRC (more inclusion of CLK1-E4, COL6A3-E6, CD44v8–10, alternative first exon regulation of ARHGEF9, CHEK1, HKDC1 and HNF4A) or metastasis (decrease of SERPINA1-E1a, CALD-E5b, E6). Except for CHEK1, all other 8 splicing events were confirmed by TCGA data with 382 CRC tumors and 51 normal controls. The combination of three splicing events was used to build a logistic regression model that can predict sample type (CRC or normal) with near perfect performance (AUC = 1). Two splicing events (COL6A3 and HKDC1) were found to be significantly associated with patient overall survival. The AS features of the 9 genes are highly consistent with previous reports and/or relevant to cancer biology.

**Conclusions:**

The significant association of higher expression of the COL6A3 E5-E6 junction and HKDC1 E1-E2 with better overall survival was firstly reported. This study might be of significant value in the future biomarker, prognosis marker and therapeutics development of CRC.

## Background

Colorectal cancer (CRC) is the third and second most common cancer in men and women worldwide [1] and it’s the second and third leading cause of death in men and women in developed countries [1]. A deep understanding of the genes involved in the tumorigenesis of CRC will eventually contribute to developing diagnosis, prognosis and therapeutic methods of CRC.

AS is an important mechanism of regulating eukaryotic gene expression. Ninety-two to 94 % of human genes undergo AS. Different protein isoforms produced by the same gene through AS may have related, distinct or even opposing functions [2]. Regulation of AS plays an important role in both normal and disease states of biological processes [3, 4]. In cancer research, defects of AS or mutation, misregulation of splicing factors were linked to tumorigenesis [5, 6], cancer metastasis [7] and cancer drug resistance [8]. Targeting AS or targeting splicing factors are new therapeutic approaches to fight against cancer [9–13].

Aberrant splicing events in CRC and/or other cancer types were identified using either microarray or RNA-seq techniques in the past [14–17]. The roles of AS in CRC were reported for individual genes: ITGA6 [18], MAP4K4 [19], EPDR1, ZNF518B [20], FIR [21], BRAF [22], Rac1 [23], OCT4 [24], RON [25], CD44 [26, 27], KRAS [28], ZNF148 [29], FAK [30]. Global AS analysis in CRC or Pan-Cancer has also been performed using large number of RNA-seq data (i.e. TCGA) with focuses on different aspects, such as AS in genes related to energy metabolism [31], impacts on protein function [32] and neoantigens [33].

In this study, we sought to identify recurrent AS event as potential biomarkers for diagnosis, prognosis and potential new therapeutic targets in CRC. With the publicly available RNA-seq data, we studied 28 pairs of CRC and normal tissues and 18 pairs of metastatic and normal tissues. As results, we identified AS events of 9 genes in CRC or metastasis. Eight splicing events were confirmed by independent TCGA data with 382 CRC tumors and 52 normal controls, which could be used as biomarkers of CRC. One AS event in CLK1 gene might be a novel therapeutic target. Finally, two AS events were found to be significantly associated with patient overall survival.

## Methods

### RNA-seq datasets

We collected two public RNA-seq data related to CRC from GEO database (Supplementary Table 1): GSE50760 [34] contains normal colon (NC), primary CRC (CRC), and liver metastasis (MC) for 18 patients. GSE95132 [35] contains 10 pairs of primary tumors (CRC) and matching normal colon tissues (NC). Based on the number of number of patients in the two datasets, we named the two datasets CRC18P and CRC10P respectively.

### RNA-seq data analysis

Raw reads in fastq format of the two GEO datasets (Supplementary Table 1) were downloaded from SRA (https://www.ncbi.nlm.nih.gov/sra/) using SRA Toolkit. Reads were mapped to the human hg19 genome using STAR (2.5.3a) [36]. Only the uniquely mapped reads with MAPQ value > 10 and mismatch < 5% of the read length were used.

The Sashimi Plot showing the junction read number was generated using Integrative Genomics Viewer (IGV) [37]. The uniquely mapped reads mapping from bam files were converted to bedgraph format using the genomeCoverageBed command (from bedtools, https://bedtools.readthedocs.io/en/latest/index.html). Instead of visualizing read number coverage, all read coverage data in the bedgraph file were normalized to reads per million (RPM) value by dividing by the total number uniquely mapped reads in a sample. Then the bedgraph files were converted to bigwig file using the bedGraphToBigWig program downloaded from UCSC genome browser (http://hgdownload.soe.ucsc.edu/admin/exe/) and visualized in UCSC genome browser (https://genome.ucsc.edu). The height of each coordinate in the genome represents the RPM value of the read coverage at that coordinate.

For splicing analysis of exons, we used Refseq defined exons and counted the reads mapped to them using custom perl and R scripts (available at https://github.com/tianjiagene/GneneBlockReadCount). The exon read counts table were used as input for the R library DEXSeq [38]. The adjusted *P*-value< 0.001 and fold change> 2 were used to select significantly regulated exons.

### Percent-spliced-in (PSI) analysis

**Fig. 1:**
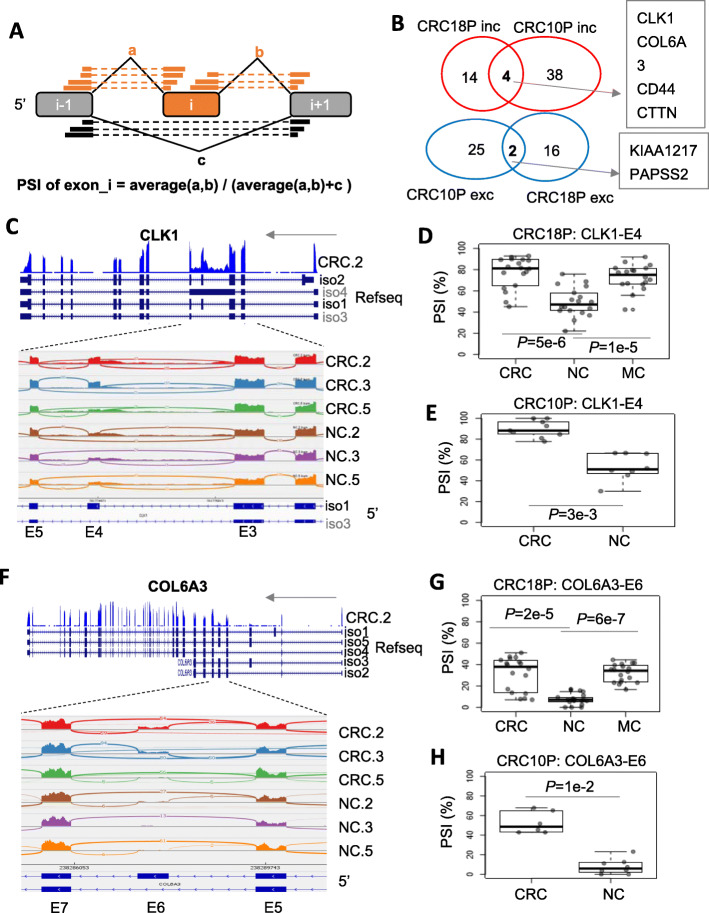
Cassette exon regulation in CRC. **a** Diagram of the method to calculate the PSI of an exon. The orange boxes and gray boxes are the alternative exons and neighboring exons. Thick bars connected by a dotted line represent a read cover two exons (junction read). a, b and c are read counts for the three junctions. **b** Venn diagram showing the overlap of exon splicing events between CRC and NC in CRC18P and CRC10P datasets. *P*-value of Wilcoxon rank-sum test< 0.05 and |ΔPSI| > 20% were used as cutoffs to select significant events. Inc., exon inclusion in CRC; Exc, exon exclusion in CRC. **c** CLK1 gene structure (top) and read coverage (Sashimi Plot) for exon 3 to exon 5 region. **d** Boxplot of PSI of CLK1 exon4 in the CRC18P dataset. Dots in the boxplot represent individual patient data. *P*-value is based on Wilcoxon rank-sum test. **e** Similar to (**d**) except that the CRC10P dataset was shown. **f** COL6A3 gene structure (top) and read coverage (Sashimi Plot) for exon 5 to exon 7 regions. **g** Boxplot of PSI of COL6A3 exon6 in the CRC18P dataset. Dots in the boxplot represent individual patient data. *P*-value is based on Wilcoxon rank-sum test. **h** Similar to (**g**) except that the CRC10P dataset was shown

**Fig. 2:**
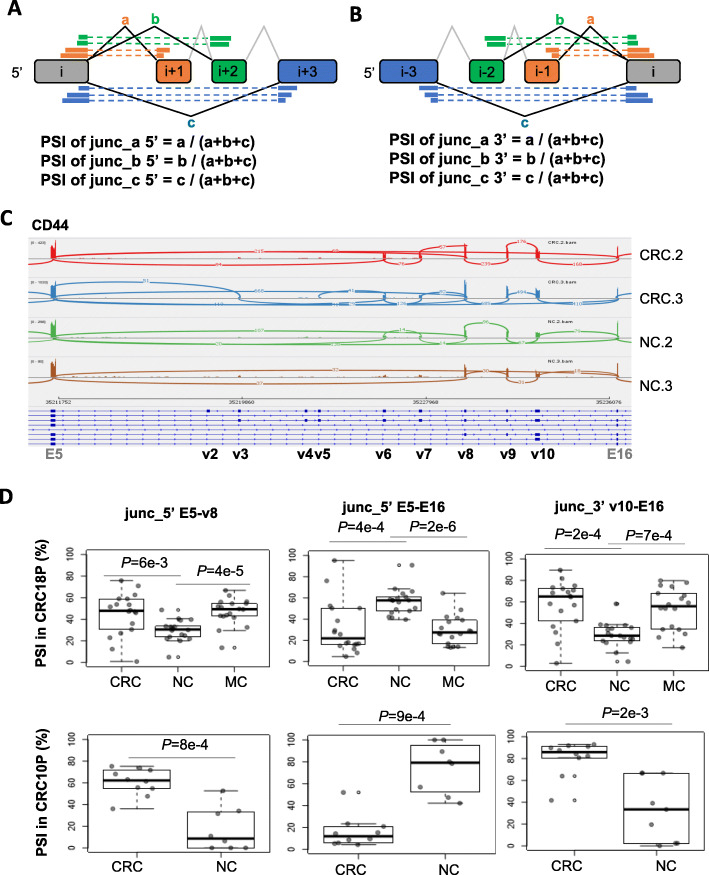
CD44v8–10 showed up-regulation in CRC at the expense of other CD44 splicing variants. **a** Diagram of the method to calculate PSI_junc5’, which represents the usage of a junction among all junctions sharing the same 5’ splice site. The boxes are exons. Thick bars connected by a dotted line represent a read cover two exons (junction read). a, b and c are read counts for the three junctions. **b** Similar to (**a**) except that diagram of PSI_junc3’ was shown, which represents the usage of a junction among all junctions sharing the same 3’ splice site. **c** CD44 gene structure (bottom) and read coverage (Sashimi Plot) for exon 5 to exon 16 regions. **d** Boxplot of PSI of CD44 junc_5’ E5-v8 (top row), junc_5’ E5-E16 (middle row) and junc_3’ v10-E16 (bottom row) in CRC18P (left column) and CRC10P (right column) datasets. Dots in the boxplot represent individual patient data. *P*-value is based on Wilcoxon rank-sum test

We used three PSI calculation methods: PSI_exon, PSI_junc5′ and PSI_junc3′ as illustrated in Fig. 1A, Fig. 2A, and B, respectively. We required a minimal total read of 10 to calculate a PSI value. The PSI values of two groups of samples (eg. CRC vs. NC) were compared using Wilcoxon rank-sum test. For ΔPSI (the PSI change between two sample groups), the PSI values of each group were averaged and then the difference was calculated. *P*-value < 0.05 and |ΔPSI| > 20% were used as cutoffs to select significant AS events if not mentioned. All statistical analysis and plots were performed using R.

### TCGA data and analysis

All TCGA patient overall survival (OS) data and junction usage data were downloaded from TSVdb [39]. We selected 382 CRC tumors samples (379 primary solid tumor samples, 1 metastatic and 2 recurrent solid tumor samples) and 51 normal colon or rectal tissue samples and downloaded data for the 9 genes identified from the AS analysis. For survival analysis, all patients with OS data were separated into two groups based on the median value of a particular junction usage. The survival curve was drawn using the R package “survminer” (https://cran.r-project.org/web/packages/survminer/). *P*-value is calculated based on the log-rank test. The data for percent of stromal cells in tumor tissues were downloaded from the GDC website (https://portal.gdc.cancer.gov/, slide.tsv file, percent_stromal_cells column). The correlation of junction usage and percent of stromal cells was tested using Spearman correlation.

### Logistic regression analysis of TCGA data

The junction usage data for the eight genes in 382 CRC tumors samples (379 primary solid tumor samples, 1 metastatic and 2 recurrent solid tumor samples) and 51 normal colon or rectal tissue samples were used to train and predict whether a sample is a CRC or normal. Each time, up to 3 junctions/genes were selected as predictors. A logistic regression model was built based on randomly selected 50% of the samples. Then the model was used to predict the rest 50% of the samples. The Area Under Curve (AUC) of Receiver Operating Characteristic (ROC) curve was used as indicator of how good the model is. For the same set of predictors, this training and testing process was performed 5 times. The average of the 5 AUC scores was used to evaluate the overall performance of the set of predictors. Among the 5 models in the best performed predictor set, the model with the top AUC score was picked as the final model.

## Results

### Identification of commonly regulated alternative exon splicing events from RNA-seq data using multiple CRC sample cohorts

We collected two public RNA-seq data related to CRC from GEO database (Supplementary Table 1): CRC18P and CRC10P, in which paired tumor (CRC) and normal colon tissue (NC) were collected from 18 and 10 patients, respectively. We applied percent-spliced-in (PSI) analysis (Fig. 1a, see Methods for details) to identify splicing events, which are different in CRC compared to NC tissues with PSI change> 20% and *P* < 0.05. Overall the ∆PSI values are correlated between CRC18P and CRC10P cohorts (supplementary Figure 10A). As results, six exons showed significant regulation in both CRC18P and CRC10P cohorts. Three exons in three genes (CLK1, COL6A3 and CD44) showed relatively more magnitude of PSI change among the six and were followed up in more detail (Fig. 1b, Supplementary Table 2–3).

The exon 4 of CLK1 has a median inclusion level (PSI) of about 50% in NC samples and higher inclusion level (median 80–90%) in CRC samples in CRC18P and CRC10P (Fig. 1c-e, supplementary Figure 1). In CRC18P, MC samples maintain the higher PSI compared to NCs (Fig. 1d).

The exon 6 of COL6A3 has a median inclusion level (PSI) of about 10% in NC samples and higher inclusion level (median 40–50%) in CRC samples in CRC18P and CRC10P (Fig. 1f-h, supplementary Figure 2). In CRC18P, MC samples maintain the higher PSIs as CRCs compared to NCs (Fig. 1g).

### Complex AS of CD44 gene in CRC

Exon v10 of the CD44 gene was found to be upregulated in CRC compared to NCs (Fig. 1b, Supplementary Table 2-3, supplementary Figure 3). Exon v10 is the last exon of the 9 alternative exons between exon 5 and exon 16 (Fig. 2c). We noticed multiple alternative exons were included in CRC tissues. The complexity of the AS in CD44 prompted us to use a modified PSI calculation method. As shown in Fig. 2a-b, PSI_junc5′ represents the usage/expression of an exon-exon junction compared to all junctions sharing the same 5′ splice site. PSI_junc3′ represents the usage/expression of an exon-exon junction compared to all junctions sharing the same 3’ splice site. Results showed an increased expression of junc5’_E5-v8 (Fig. 2d left), junc3’_v10-E16 (Fig. 2d right), and decreased expression of junc5’_E5-E16 (Fig. 2d middle) in CRC tissues in both cohorts. All other junctions showed less significant changes (data not shown). These data indicate an increase of CD44v8–10 (inclusion of exons v8, v9, and v10) and the decrease of CD44s (standard isoform that skips all 9 alternative exons) isoform in CRC tissues. Metastatic tissues also showed similar changes compared to NCs (Fig. 2d top).

### Alternative first exon regulation in CRC

**Fig. 3:**
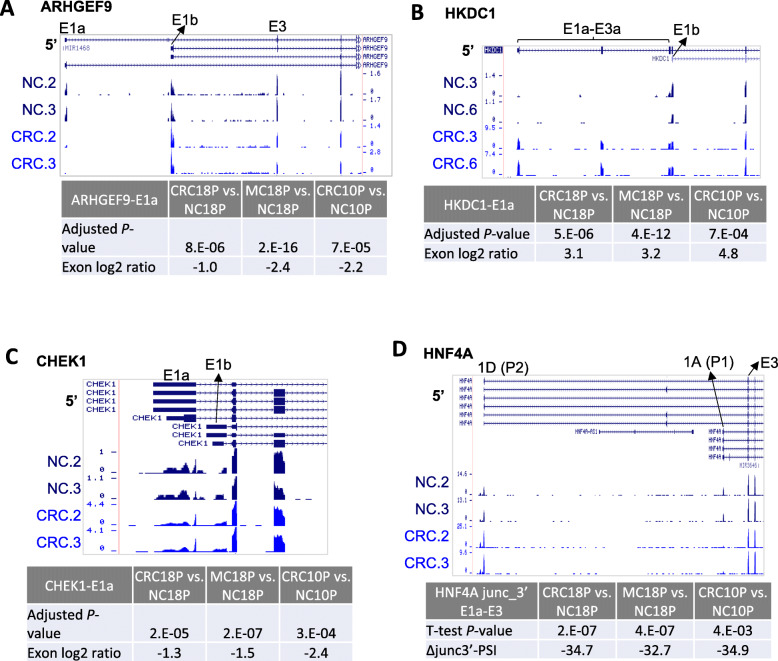
Alternative first exon regulation in CRC. **a** Read coverage of ARHGEF9 alternative first exons E1a and E1b. The height of the RNA-seq tracks represents the Read Per Million (RPM) values of the read coverage at each genomic location. The adjusted *P*-value and the log2 ratio (based on DEXSeq) of E1a were shown. **b** Similar to (**a**) except that gene HKDC1 was shown. **c** Similar to (**a**) except that gene CHEK1 was shown. **d** Similar to (**a**) except that gene HNF4A was shown and Wilcoxon rank-sum test *P*-value and ΔPSI of junc_3’ E1a-E3 was shown

Alternative terminal exon regulations including alternative first exons or alternative last exons are types of AS, which couples with alternative transcription start site and alternative polyadenylation, respectively. Regular method of calculating PSI for an exon (Fig. 1a) does not apply to these situations since only one side of the terminal exon has splice junctions. We used DEXSeq [38], PSI_junc5’ and PSI_junc3’ (Fig. 2a-b) to study these events. The basic criteria for DEXSeq data are adjusted *P*-value< 0.001 and fold change> 2 (Supplementary Table 2, 4). The criteria for PSI_junc5’ and PSI_junc3’data are Wilcoxon rank-sum test P-value< 0.05 and PSI change> 20% (Supplementary Table 2, 5–6). After manual inspection of the raw results, we finally identified four events (Fig. 3a-d, supplementary Figure 4, 5, 6, 7) regulated in CRC compared to NC. All the four events are alternative first exon events. ARHGEF9 showed down-regulation of the more upstream first exon E1a (significant in both DEXSeq and PSI_junc3’ methods, Fig. 3a, supplementary Figure 11A). HKDC1 E1a-E3a showed up-regulation (significant in DEXSeq method only, Fig. 3b). CHEK1 first exon E1a also showed downregulation (Fig. 3c, significant in DEXSeq method only). HNF4A exon 1A (more downstream than 1D) showed downregulation, relative to exon 1D (significant in PSI_junc3’ method only, Fig. 3d, supplementary Figure 11B). In all the four cases, changes of the first exon will alter the protein sequences thus will have a potential functional impact on the genes.

### AS associated with metastasis of CRC

**Fig. 4:**
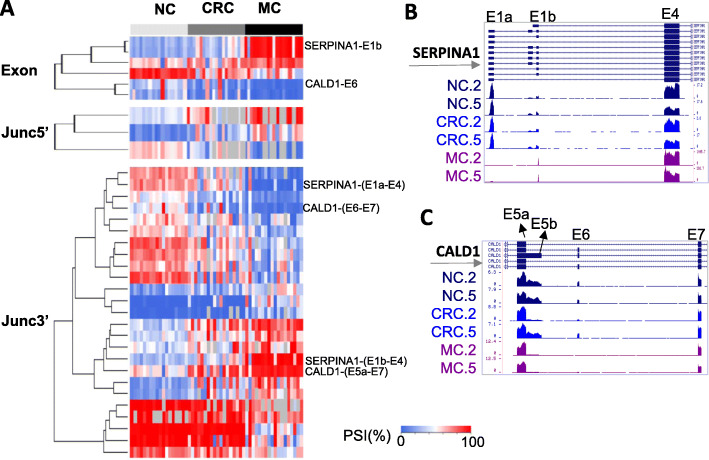
Metastasis-related splicing events. **a** Heat map showing the PSI exon, PSI_junc5’ and PSI_junc3’ values for metastasis-related splicing events. Several exons or junctions were labeled on the right. **b**-**c** Read coverage for SERPINA1 alternative first exons (**b**) and CALD1 E5 to E7 (**c**)

Taking the advantage of the metastasis data from CRC18P, we sought to identify splicing events associated with metastasis of CRC. The ∆PSI values are correlated between CRC (vs. NC) and MC (vs. NC) in CRC18P cohort (supplementary Figure 10B). We selected a subset of splicing events with the further increase or decrease of PSI values in metastatic tissues compared to CRC by at least 15%. The following criteria were used to select these events: MC vs. NC |ΔPSI| > 20% and *P* < 0.001 (Wilcoxon rank-sum test), and MC vs. NC and CRC vs. NC in CRC18P and CRC10P data showing a consistent trend (same sign). 6, 3 and 25 events were identified from PSI_exon, PSI_junc5’ and PSI_junc3’ data, respectively (Fig. 4a, Supplementary Table 2–3, 5, 6). We manually selected two splicing events in SERPINA1 and CALD1 based on the fact that they were picked up by multiple analysis methods. The results indicated a higher magnitude of PSI change in metastatic samples (Fig. 4a-c). In metastatic samples, the first exon of SERPINA1 switched from 1a to more downstream one 1b (Fig. 4b, supplementary Figure. 8, 11C). CALD1 tended to express a shorter version of exon 5 and had exon 6 skipped by alternative 5’ splice site usage and exon skipping respectively (Fig. 4c, supplementary Figure 9, 11C).

### Validation of splicing events using TCGA data

**Fig. 5:**
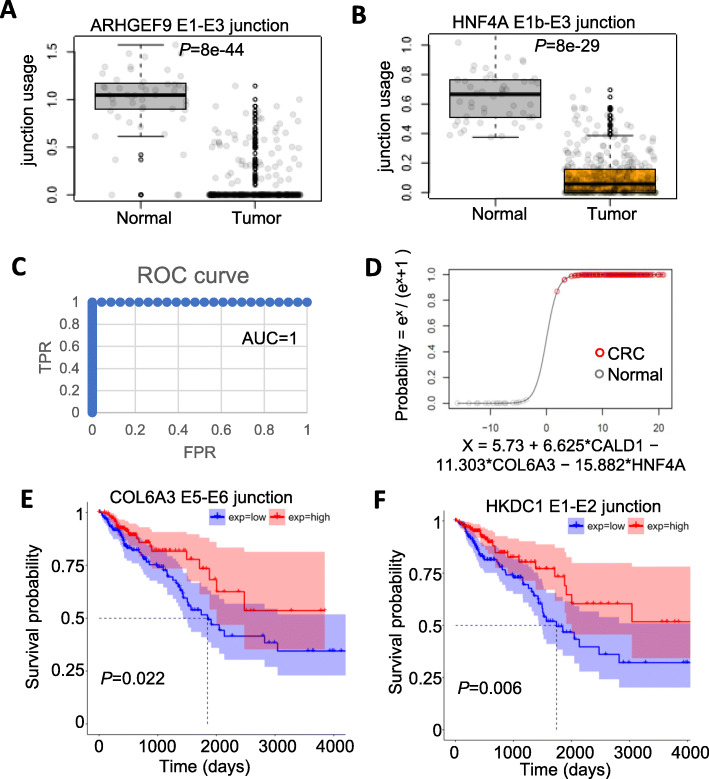
Splicing events validated by TCGA data and potential value in cancer diagnosis and overall survival (OS)**. a**-**b** Boxplots of junction usage of ARHGEF9 E1-E3 junction (**a**) and HNF4A E1b-E3 junction (**b**) in 51 normal tissue and 382 CRC or metastatic tissue (tumor). *P*-values are based on Wilcoxon rank-sum test adjusted using Bonferroni correction. Dots in the boxplot represent the individual patient in TCGA. **c** Receiver operating characteristic (ROC) curve of a logistic regression model using junction usages from three genes (CALD1, COL6A3, HNF4A) as predictors and sample type (normal, value = 0 or CRC, value = 1) as the dependent variable. Area Under Curve (AUC) is shown. **d** Logistic regression curve of the model as shown in C. X axis is the logit (log odds) function. Y axis is the predicted probability of the sample type. Only the testing data (not used in the training process) were used in the plot. The actual sample types were shown as red and gray circles for CRC and normal respectively. **e**-**f** Survival curves of 357 patients with overall survival data equally separated into two groups (low and high) based on junction usage of COL6A3 E5-E6 (**e**) and HKDC1 E1-E2 (**f**). *P*-value is based on the log-rank test. Confidence intervals were shown as shaded areas

To validate the splicing events identified in this study in an independent dataset, we used a recently developed tool TSVdb [39], which integrates and visualizes AS data based on TCGA samples for 33 tumor types. We searched all the 9 genes identified in this study (CLK1, COL6A3, CD44, ARHGEF9, HKDC1, CHEK1, HNF4A, SERPINA1, CALD1) against TSVdb and downloaded the junction usage value for colon adenocarcinoma (COAD) and rectum adenocarcinoma (READ). Junction usage is calculated by dividing junction quantification value to mean junction quantification value of that person for a specific gene [39]. We combined junction usage for 379 primary solid tumor samples with 1 metastatic and 2 recurrent solid tumor samples (382 CRC tumors samples in total) and compared with 51 normal colon or rectal tissue samples. Results indicated that, except for one gene CHEK1 (not significant, nominal *P* = 0.2), all other 8 genes showed significant splicing changes in tumors samples compared to normal tissues (Wilcoxon test, Bonferroni adjusted *P*-value range from 2 × 10^− 15^ to 8 × 10^− 44^, supplementary Figure 12, supplementary Table 2, 7), consistent with what we observed in the two GEO datasets. ARHGEF9 E1-E3 junction and HNF4A E1b-E3 junction showed most significant *P*-values (Fig. 5a-b). The highly consistent results among several cohorts of patient data indicate that the splicing events identified in this study are reliable markers of CRCs.

TCGA tumor samples may contain a fraction of stromal cells which may confound the splicing analysis. We tested the correlation of junction usage and percent of stromal cells for 382 CRC or metastatic tissues for the junctions from the 9 genes. Most junctions do not show any correlation with percent of stromal cells except for a junction in CD44 (Rho = − 0.17, Bonferroni corrected *P* = 0.013), in which high stroma content is associated with lower expression of the isoform that is more highly expressed in tumors (Supplementary Table 2, 8). These indicated that stromal content in general does not confound the splicing analysis but in some cases, it may compromise the analysis.

To test whether the splicing events for multiple genes identified from this study can be used as biomarkers in CRC, we performed logistic regression analysis. We selected the 8 genes with significant junction usage in CRC compared to normal (supplementary Figure 12, supplementary Table 2, 7). For each gene, only one junction with the most significant P-value was selected. The resulting 8 junctions from 8 genes were separated to 3 groups based on the correlation of their junction usage in all samples (supplementary Figure 13A). The group 1 is more distinct from group 2 and 3. To build a logistic model, we tried to identify the best combination of junctions/genes as predictors. We performed the analysis using single predictors (using the 8 genes separately), two predictors (using one from group 1 and 1 from group 2 or 3) and three predictors (using one from each of the three groups). In total, 41 sets of predictors were evaluated. Whether the sample is a CRC (value = 1) or normal (value = 0) is the dependent variable in the model. For each predictor set, 5 times of training and testing process were performed with each time using randomly selected 50% of the samples for training and the rest of samples for testing. The average AUC scores range from 0.873 to 0.999 (supplementary Figure 13B). The models using more predictors perform better than less predictors but it’s not always the case. 17 of the 41 predictor sets have an average AUC above 0.99 including 7 two-predictor sets and 10 three-predictor sets. The best predictor set is the one using three junctions from CALD1, COL6A3 and HNF4A. 3 of the 5 models using this set of predictors has an AUC score of 1 (100% true positive and 0% false positive rate). One of the 3 models is shown in Fig. 5c (ROC curve) and D (logistic regression curve) using the testing data only.

### Relevance of splicing events to the survival of patients

To assess the clinical significance of these findings, we compared the overall survival (OS) data for patients with different splicing profile for the 10 genes. For each junction usage data, we separated all of patients with OS data into two equal sized groups (high and low junction usage groups). We found that two junctions (COL6A3 E5-E6 junction and HKDC1 E1-E2 junction) showed a significant difference in OS (Fig. 5e-f). The patients with higher expression of both junctions, which are also more expressed in CRC cancers (Figure 1g h and Fig. 3b), are associated with better survival.

## Discussion

Global splicing changes in cancer have been studied using microarray or RNA-seq techniques in the past [14–16]. Compared to microarray (eg. Affymatrix Exon Array), RNA-seq can more precisely measure the splicing changes because the reads covering the exon-exon junction directly represent the splicing choice. Usually, a PSI value can be calculated based on the read counts of the upstream junction, downstream junction and the junction skipping the exon (eg. Fig. 1a). In this study, we used two additional approaches (PSI_junc5’ and PSI_junc3’) to calculate the PSI values (Fig. 2a-b). The two approaches have the advantage to deal with more complex splicing events like in CD44, nine consecutive alternative exons can be partially included, or all excluded. Using regular PSI exon method, only exon v10 was found to be significantly included in CRC. However, combining with the two additional PSI analysis, we concluded that the isoform CD44v8–10 is upregulated in CRC. The PSI_junc5’ and PSI_junc3’ approaches can also be used to study alternative last exons and alternative first exons, respectively. In this study, we identified 5 alternative first exon events in CRC or metastasis tissues. Except for HNF4A, none of the other four events was reported previously. The results indicated that the new approaches could identify novel splicing events. Interestingly, using the same approach, we did not identify any significant alternative last exon events. This may indicate that the transcription-coupled alternative first exon choices play a more important role in CRC compared to alternative polyadenylation-coupled last exon choices.

The exon4-skipping isoform of CLK1, predominantly expressed in NC samples, matches the isoform 3 transcript from Refseq annotation. Interestingly, isoform 3 is annotated as a non-coding transcript because skipping the 91 nt exon 4 is predicted to cause out-of-frame translation and the nonsense mediated decay (NMD) of the transcript (Fig. 1c). The increase of exon4-inclusion isoform (isoform 1) at the expense of isoform 3 in CRC and MC may thus increase the productive transcript level of CLK1 and potentially produce more proteins. CLK1 encodes a member of the CDC2-like family of protein kinases (CLKs). In the cell nucleus, CLKs phosphorylate serine/arginine-rich proteins, release them into the nucleoplasm and then regulate AS of genes. Small molecule inhibitors against these CLKs were developed and exhibited growth suppression, apoptosis induction and anti-tumor effect [40, 41]. Here, we found that CLK1 itself can be alternatively spliced. CRC cells may increase the CLK1 expression by regulating the inclusion of exon 4. Other CLKs (CLK2, CLK3 and CLK4) are also expressed in CRC and normal tissues from the RNA-seq data we analyzed. It’s unclear whether the CLK1 splicing change in CRC have a major functional impact of CLKs or not. If the function of this splicing event can be validated by further evidences, exon 4 skipping could be a new target for cancer therapy.

The splicing change of the exon 6 of COL6A3 has been reported previously in colon, bladder, prostate and pancreatic cancers tissues compared to normal tissues [14, 42], indicating that the splicing change may play a role in multiple cancer types. High expression of COL6A3 in stroma were associated with poor prognosis in CRC [43]. The percent of stromal cells does not correlate with the junction usage of COL6A3 (Supplementary Table 2, 8), excluding the possibility that the splicing events is caused by the variability of stroma content in tumor tissues. COL6A3 was also found to be a key hub gene in the cell migration/extracellular matrix module that was associated with poor prognosis in CRC [44]. COL6A3 knockout decreases cell proliferation and invasion, increases cell apoptosis in cancer cell lines [44]. Taken together, the relevance and importance of COL6A3 to CRC tumorigenesis were highlighted. Our finding of exon 6 splicing change added more complexity of the role of COL6A3 in CRC and this may serve as an additional biomarker in the diagnosis of CRC.

The expression of alternatively spliced CD44 adhesion molecules has been implicated in the pathogenesis and metastasis of colorectal cancer. mRNA expression, different splice isoform expression or protein isoform expression have been used in different studies. However, the results are usually conflicting. Either positive correlation [27, 45–48] or no correlation [49] to CRC or metastasis has been reported. Our study suggests that isoform CD44v8–10 is upregulated in CRC and liver metastasis tissue with relative downregulation of CD44s. However, metastasis did not show further increase of CD44v8–10 compared to CRC. Therefore, CD44v8–10 or maybe the ratio of CD44v8–10/CD44s could be used as a CRC biomarker.

We identified four genes (ARHGEF9, CHEK1, HKDC1 and HNF4A) with alternative first exon regulation in CRC. Although some studies indicated that many of these genes are linked to tumorigenesis or metastasis, the detailed functions of these splicing events in CRC have not been reported. ARHGEF9 has been shown to play a role in tumor cell migration, invasion and metastasis by linking oncogene CHD1L and Cdc42 pathway in hepatocellular carcinoma (HCC) [50]. HKDC1 encodes the hexokinase domain containing 1, which catalyzes the phosphorylation of glucose. Its high expression in hepatocarcinoma (HCC) is related to poor overall survival (OS) [51]. And it is also predicted to be a novel therapeutic target in lung cancer [52]. CHEK1 contributes to CDC25C-mediated Docetaxel resistance and can also be a therapeutic target in prostate cancer [53]. In HNF4A gene, the promoter P1 driven isoforms (expressing exon 1A) were decreased in CRCs and MCs compared to the promoter P2-driven isoforms (expressing exon 1D) (Fig. 3d, supplementary Figure 7). It was reported that P1-HNF4a is expressed primarily in the differentiated compartment of the mouse colonic crypt and P2-HNF4a in the proliferative compartment. The mice that could only produce P2-HNF4a experienced more colitis and developed more tumors than normal mice [54]. Taken together, the splicing changes of these genes identified in this study may contribute to tumorigenesis and can be better biomarkers than gene expression in CRC.

In this study, AS of two genes (SERPINA1 and CALD1) were found to be associated with metastasis. For SERPINA1, although the two splice isoforms have the same start codon in exon 4, their different 5’UTR sequences may contribute to different RNA decay or protein translation of the gene. Elevated expression of SERPINA1 has been associated with the advanced stage, lymph node metastasis, poor prognosis and shorter overall survival in CRC [55] and gastric cancer [56]. For CALD1 exon 6 skipping, the same splicing event was reported to be present in colon, bladder and prostate tumors compared to normal tissues [14]. The isoform expressed in metastatic CRC samples matches the low-molecular-weight isoforms (L-CAD), specifically encoded by WI-38 L-CAD II (transcript variant 2). The same isoform was significantly associated with poorer prognosis in urothelial bladder carcinoma (BC) [57]. L-CaD was also linked to lymph node metastasis and poorer prognosis in oral cavity squamous cell carcinoma (OSCC) [58].

The logistic regression analysis generated 17 models using 2 or 3 junctions as predictors with an average AUC above 0.99. This indicated that the splicing events identified here can be used as potential diagnosis biomarkers in CRC. It remains unclear whether the splicing events can be detected in circulating tumor cells. Although doing RNA-seq using patient samples is not feasible in the clinical setting, easier experimental approaches, such as RT-qPCR, NanoString, or AmpliSeq can be designed using as few as two or three genes of the 8 genes identified in this study.

We have identified two genes (COL6A3 and HKDC1) with AS associated with overall survival. The high gene expression of COL6A3 in stroma has been linked to poor overall survival in CRC [43], while high expression of gene HKDC1 is related to poor overall survival in hepatocarcinoma [51]. However, it’s still unclear whether the splicing isoforms of these two genes have the same functions. It might suggest that the splice isoforms have variable functions in different cancer types. More data is required to illustrate the functional link between the isoforms and patient survival.

Zong et al. have developed a prognostic predictors model using AS pattern of 13 genes identified from TCGA data [59]. However, normal tissue controls had not been used in that study. It’s not clear whether those splicing events are different in normal tissues.

## Conclusions

In summary, we have identified significant AS of 7 genes in CRC and its metastasis tissue, and 3 genes with the stronger effect in metastatic tissue compared to normal tissue. Among them, the more inclusion of COL6A3-E6, CD44v8–10, and the more exclusion/decrease of HNF4A P1-driven isoform, CALD-E5b and E6 have been reported in CRC previously. Other 5 splicing events were newly identified in this study. Although the splicing events of these genes were not reported previously, their gene expression level was linked to tumorigenesis or prognosis of CRC (SERPINA1) or other cancer types (ARHGEF9, CHEK1, HKDC1). Targeting the kinase activity of CLK1 was suggested to be a therapeutic approach in cancer therapy. Considering the high consistence of AS events of the 9 genes identified in this study and previously studies, as well as their high relevance to cancer, it might suggest that the splicing signature of the 9 genes could serve as diagnosis, prognosis markers and facilitate drug development of CRC as well.

## Supplementary information

**Additional file 1: Figure S1.** RNA-seq read coverage of CLK1 exon 4 and 5 (E4, E5) for all samples in CRC18P and CRC10P datasets. E4 showed more inclusion in CRC and MC samples (labeled in red).

**Additional file 2: Figure S2.** RNA-seq read coverage of COL6A3 exon 6 and 7 (E6, E7) for all samples in CRC18P and CRC10P datasets. E6 showed more inclusion in CRC and MC samples (labeled in red).

**Additional file 3: Figure S3.** RNA-seq read coverage of CD44 exon 5 to exon 16 for all samples in CRC18P and CRC10P datasets. Exons v8–10 showed more inclusion in CRC and MC samples (labeled in red).

**Additional file 4: Figure S4.** RNA-seq read coverage of ARHGEF9 alternative first exons in CRC18P and CRC10P datasets. Exon E1a showed more exclusion/downregulation in CRC and MC samples (labeled in blue).

**Additional file 5: Figure S5.** RNA-seq read coverage of HKDC1 alternative first exons in CRC18P and CRC10P datasets. Exons E1a, E2a and E3a showed more inclusion/upregulation in CRC and MC samples (labeled in red).

**Additional file 6: Figure S6.** RNA-seq read coverage of CHEK1 alternative first exons in CRC18P and CRC10P datasets. Exon E1a showed more exclusion/downregulation in CRC and MC samples (labeled in blue).

**Additional file 7: Figure S7.** RNA-seq read coverage of HNF4A alternative first exons in CRC18P and CRC10P datasets. Exon 1A showed more exclusion/downregulation in CRC and MC samples (labeled in blue).

**Additional file 8: Figure S8.** RNA-seq read coverage of SERPINA1 exon 1 to exon 4 in CRC18P dataset. Exon E1a showed more exclusion/downregulation in MC samples (labeled in blue).

**Additional file 9: Figure S9.** RNA-seq read coverage of CALD1 exon 5 to exon 7 in CRC18P dataset. Exons E5b and E6 showed more exclusion/downregulation in MC samples (labeled in blue).

**Additional file 10: Figure S10.** Scatter plot of ΔPSI_exon values. Each dot is an exon. Only exons with significant regulation (Wilcoxon rank-sum test *P*-value < 0.05 and |ΔPSI| > 20%) in either x or y axis were shown. Black dots represent events that are significant in both x and y axis. A linear regression line is based on all dots in the plot. The total number of dots (*n*), Spearman’s correlation coefficient value (ρ) and *P*-value (based on all dots) are shown.

**Additional file 11: Figure S11.** Boxplot of PSI values for genes in Figs. 4 and 5. Only AS events identified using PSI methods were plotted here.

**Additional file 12: Figure S12.** Splicing events identified in this study confirmed by TCGA junction expression data. Boxplots of junction usage of 16 junctions of 9 genes in 51 normal tissue and 382 CRC or metastatic tissue (tumor). *P*-values are based on Wilcoxon Rank-Sum Test. Nominal P-values are shown and adjusted P-values using Bonferroni correction can be found in the supplementary Table 7. Dots in the boxplot represent individual patient in TCGA.

**Additional file 13: Figure S13.** Logistic regression predicts sample type using junction usage data. (A) Spearman’s correlation coefficient of the junction usages of the TCGA data for the 8 genes. The 8 genes were separated to 3 groups (as indicated) based on the correlation values. (B) AUC scores of the ROC curves for the logistic regression using different number and combinations of the predictors. Average value of the five AUC scores for each formula were shown and the error bar is the standard deviation. Each AUC score was generated using randomly selected half of the data to train a logistic regression model and tested on the rest of the data.

**Additional file 14.**

## Data Availability

The scripts (Perl and R) used to calculate read counts mapped to Refseq-defined exons and introns are available at https://github.com/tianjiagene/GneneBlockReadCount. The two public RNA-seq data were from GEO database (accession number: GSE50760 and GSE95132).

## Abbreviations

AS: Alternative splicing
PSI: Percent-spliced-In
CRC: Colorectal cancer
NC: Normal colon
MC: Metastasis sample
TCGA: The cancer genome atlas
IGV: Integrative genomics viewer
RPM: Reads per million
OS: Overall survival
AUC: Area under curve
ROC: Receiver operating characteristic
READ: Rectum adenocarcinoma
NMD: Nonsense mediated decay
CLKs: CDC2-like family of protein kinases
HCC: Hepatocarcinoma
L-CAD: Low-molecular-weight isoforms
BC: Bladder carcinoma
OSCC: Oral cavity squamous cell carcinoma
