# Supplementary figures and images for: Identification of novel alternative splicing isoform biomarkers and their association with overall survival in colorectal cancer
Source: BMC Gastroenterol. 2020 Jun 5;20:171. doi: 10.1186/s12876-020-01288-x (PMC7275609; doi:10.1186/s12876-020-01288-x)

Figure S1.

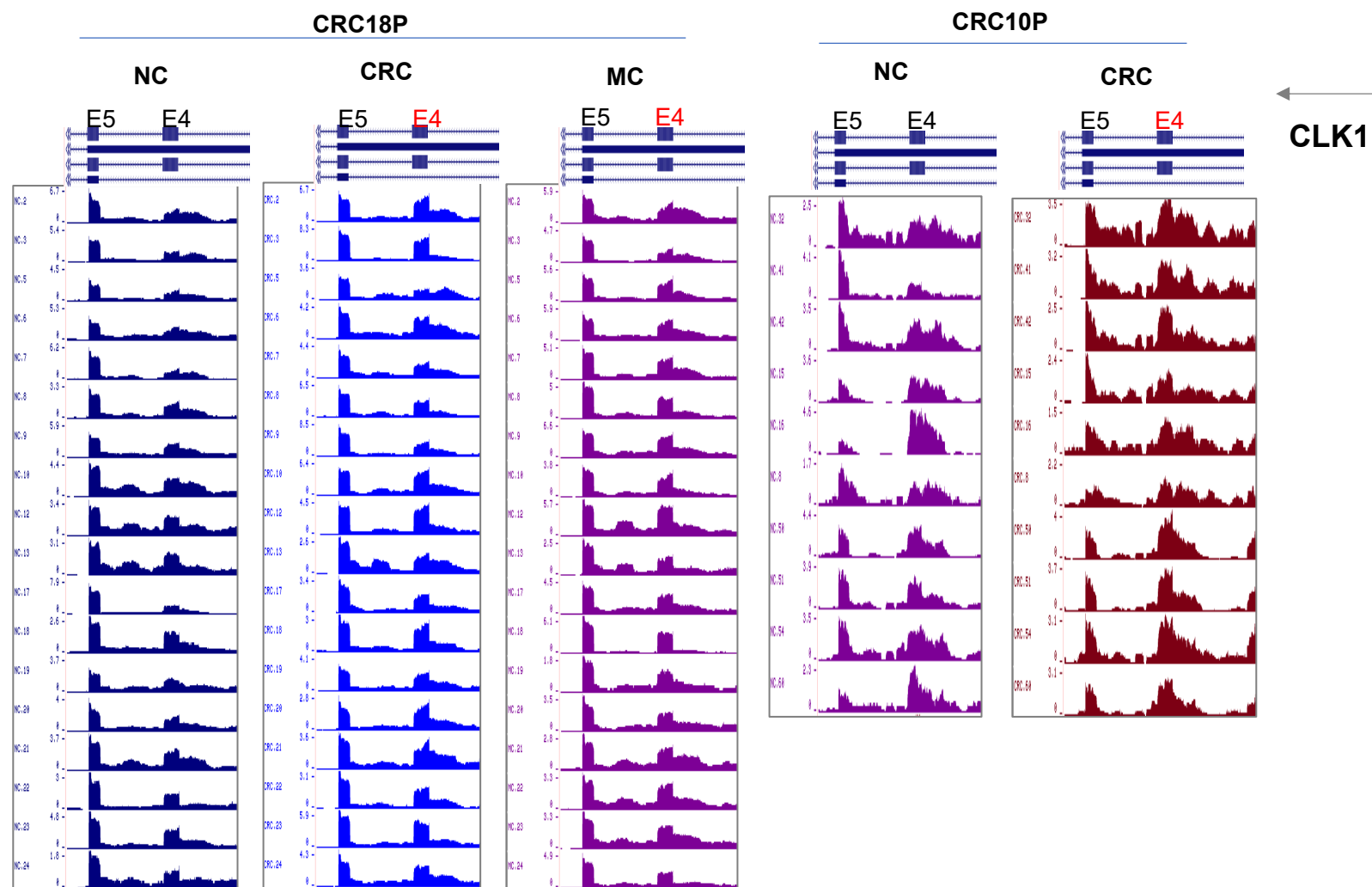

Supplement: Supplementary file 1 — Additional file 1: Figure S1. RNA-seq read coverage of CLK1 exon 4 and 5 (E4, E5) for all samples in CRC18P and CRC10P datasets. E4 showed more inclusion in CRC and MC samples (labeled in red). [file 12876_2020_1288_MOESM1_ESM.pdf]

Figure S2.

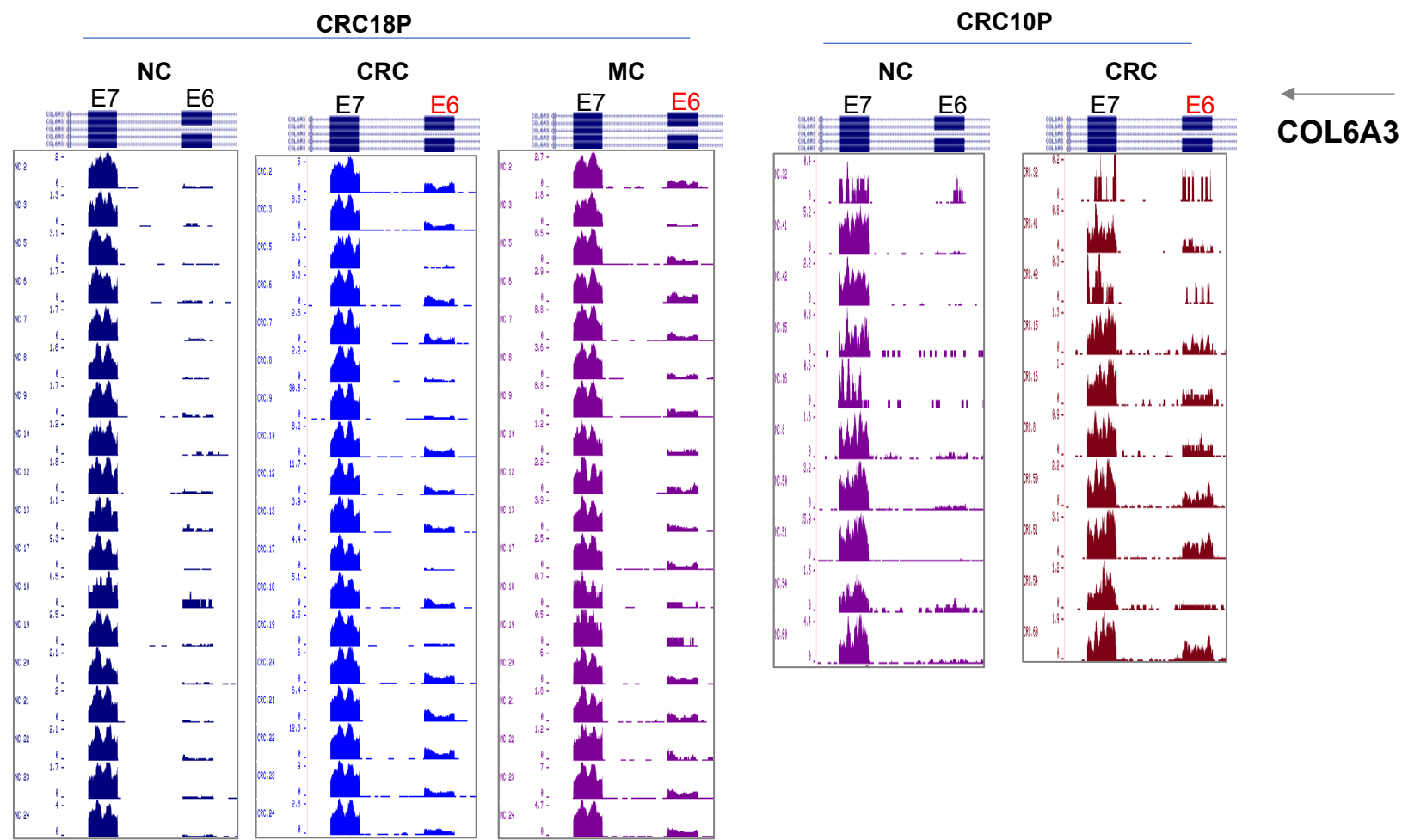

Supplement: Supplementary file 2 — Additional file 2: Figure S2. RNA-seq read coverage of COL6A3 exon 6 and 7 (E6, E7) for all samples in CRC18P and CRC10P datasets. E6 showed more inclusion in CRC and MC samples (labeled in red). [file 12876_2020_1288_MOESM2_ESM.pdf]

Figure S3.

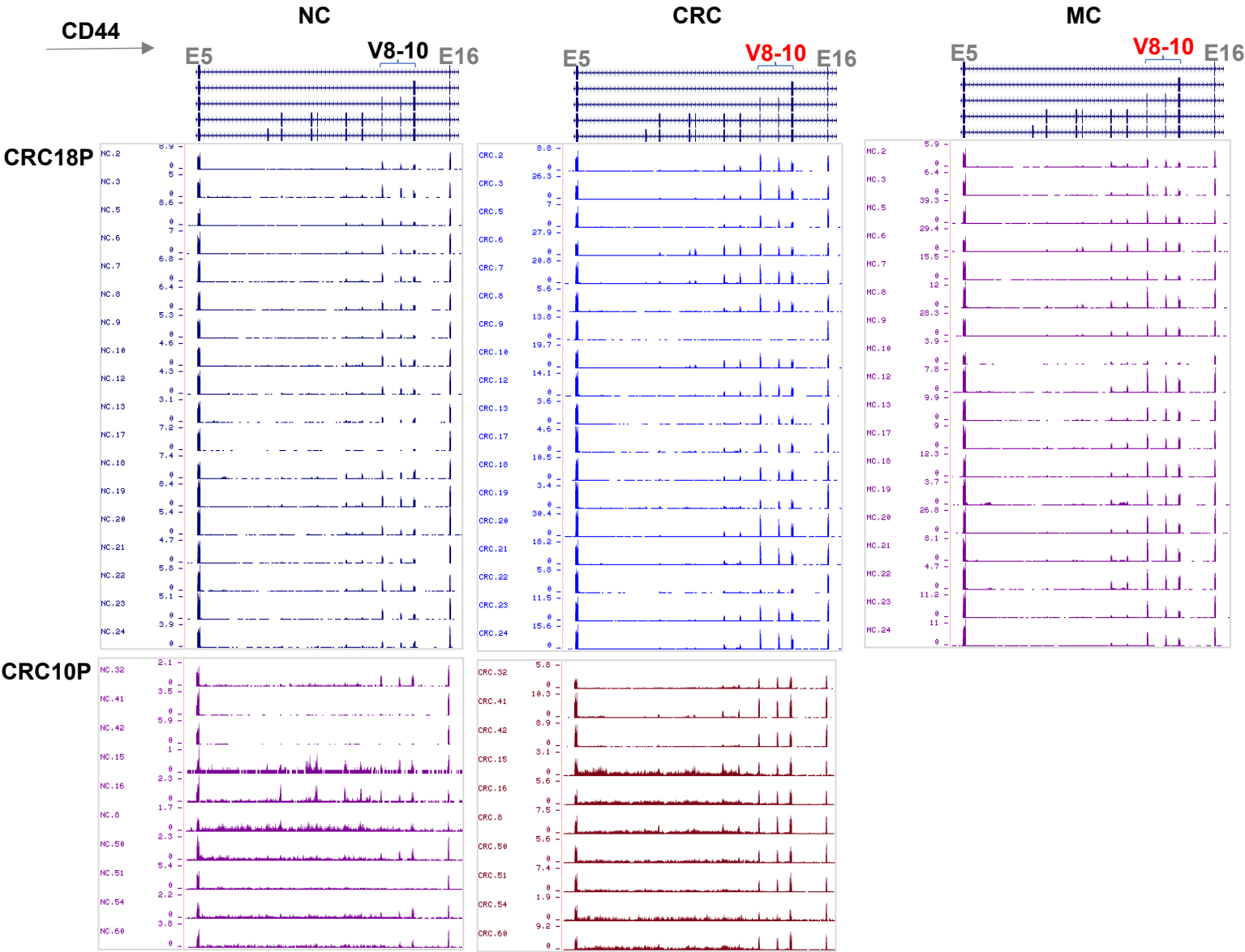

Supplement: Supplementary file 3 — Additional file 3: Figure S3. RNA-seq read coverage of CD44 exon 5 to exon 16 for all samples in CRC18P and CRC10P datasets. Exons v8–10 showed more inclusion in CRC and MC samples (labeled in red). [file 12876_2020_1288_MOESM3_ESM.pdf]

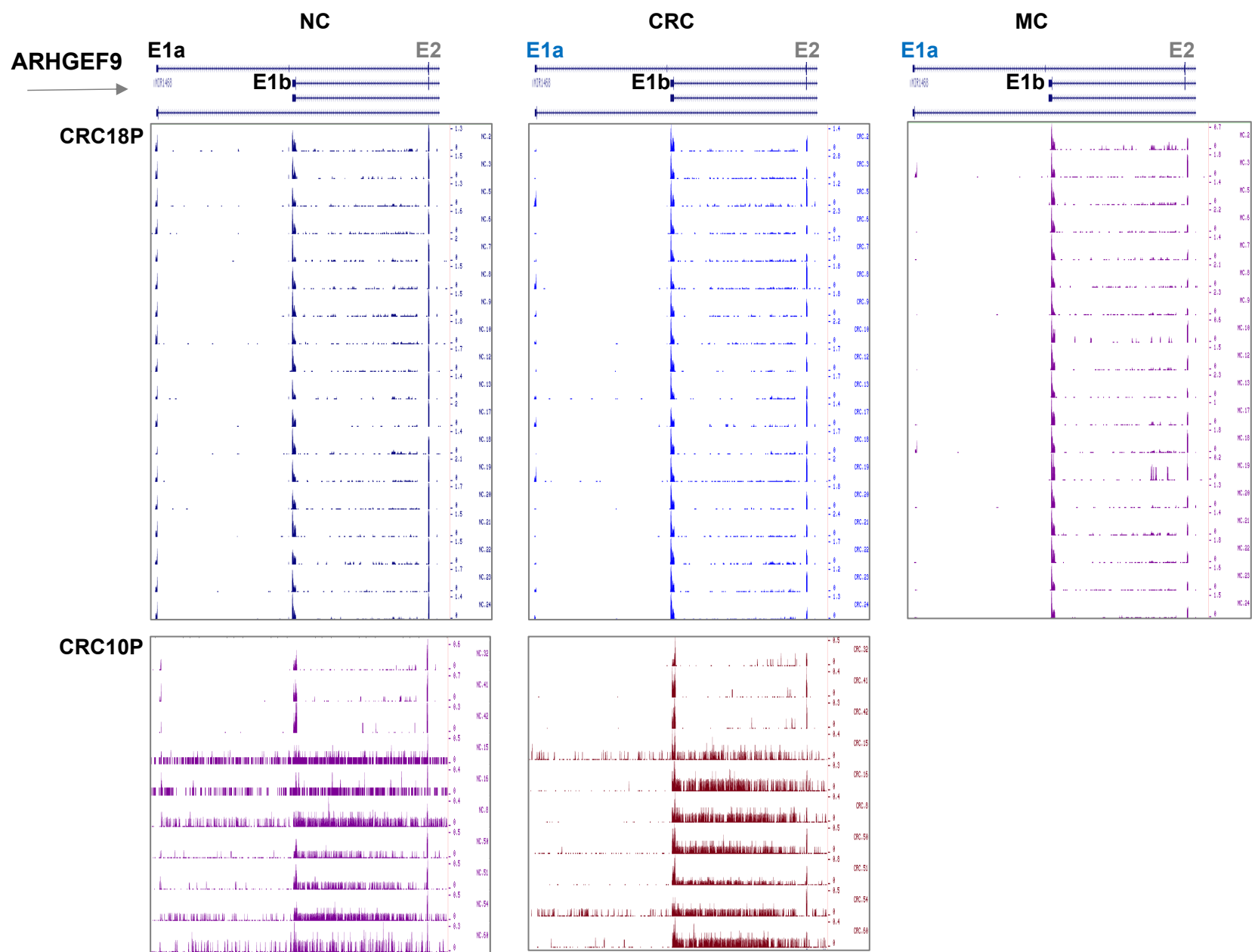

Supplement: Supplementary file 4 — Additional file 4: Figure S4. RNA-seq read coverage of ARHGEF9 alternative first exons in CRC18P and CRC10P datasets. Exon E1a showed more exclusion/downregulation in CRC and MC samples (labeled in blue). [file 12876_2020_1288_MOESM4_ESM.pdf]

Figure S5.

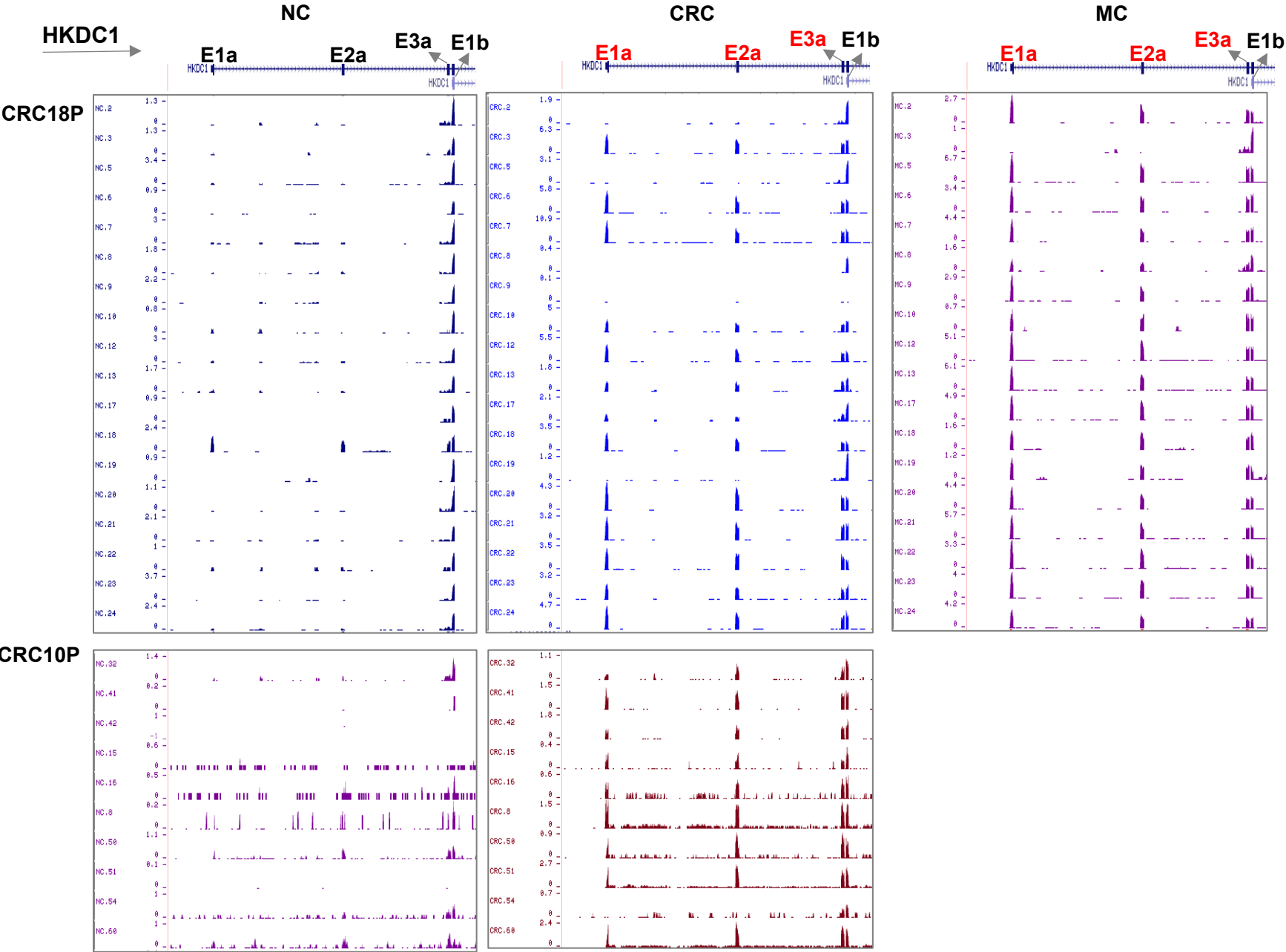

Supplement: Supplementary file 5 — Additional file 5: Figure S5. RNA-seq read coverage of HKDC1 alternative first exons in CRC18P and CRC10P datasets. Exons E1a, E2a and E3a showed more inclusion/upregulation in CRC and MC samples (labeled in red). [file 12876_2020_1288_MOESM5_ESM.pdf]

Figure S6.

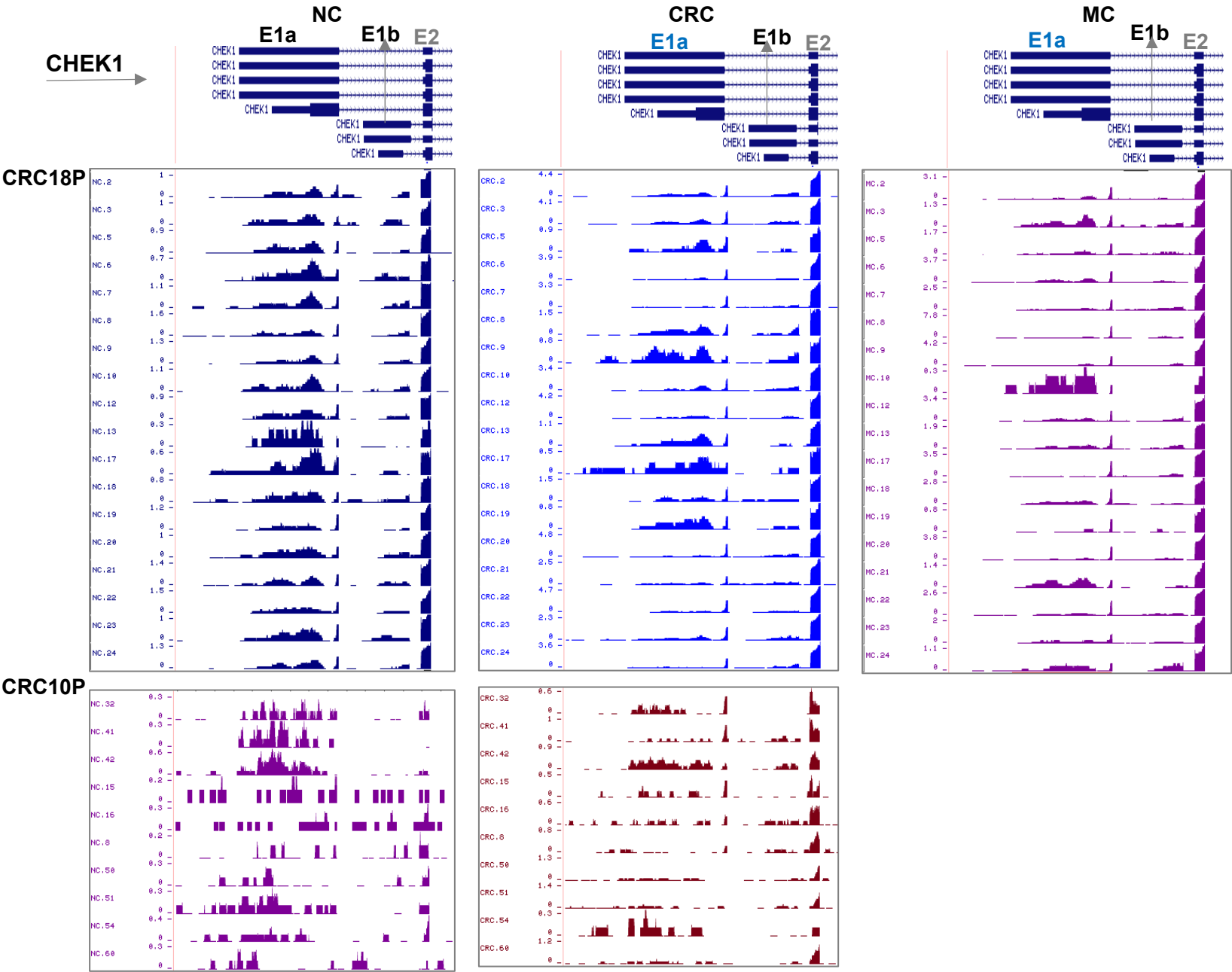

Supplement: Supplementary file 6 — Additional file 6: Figure S6. RNA-seq read coverage of CHEK1 alternative first exons in CRC18P and CRC10P datasets. Exon E1a showed more exclusion/downregulation in CRC and MC samples (labeled in blue). [file 12876_2020_1288_MOESM6_ESM.pdf]

**Figure S7.**

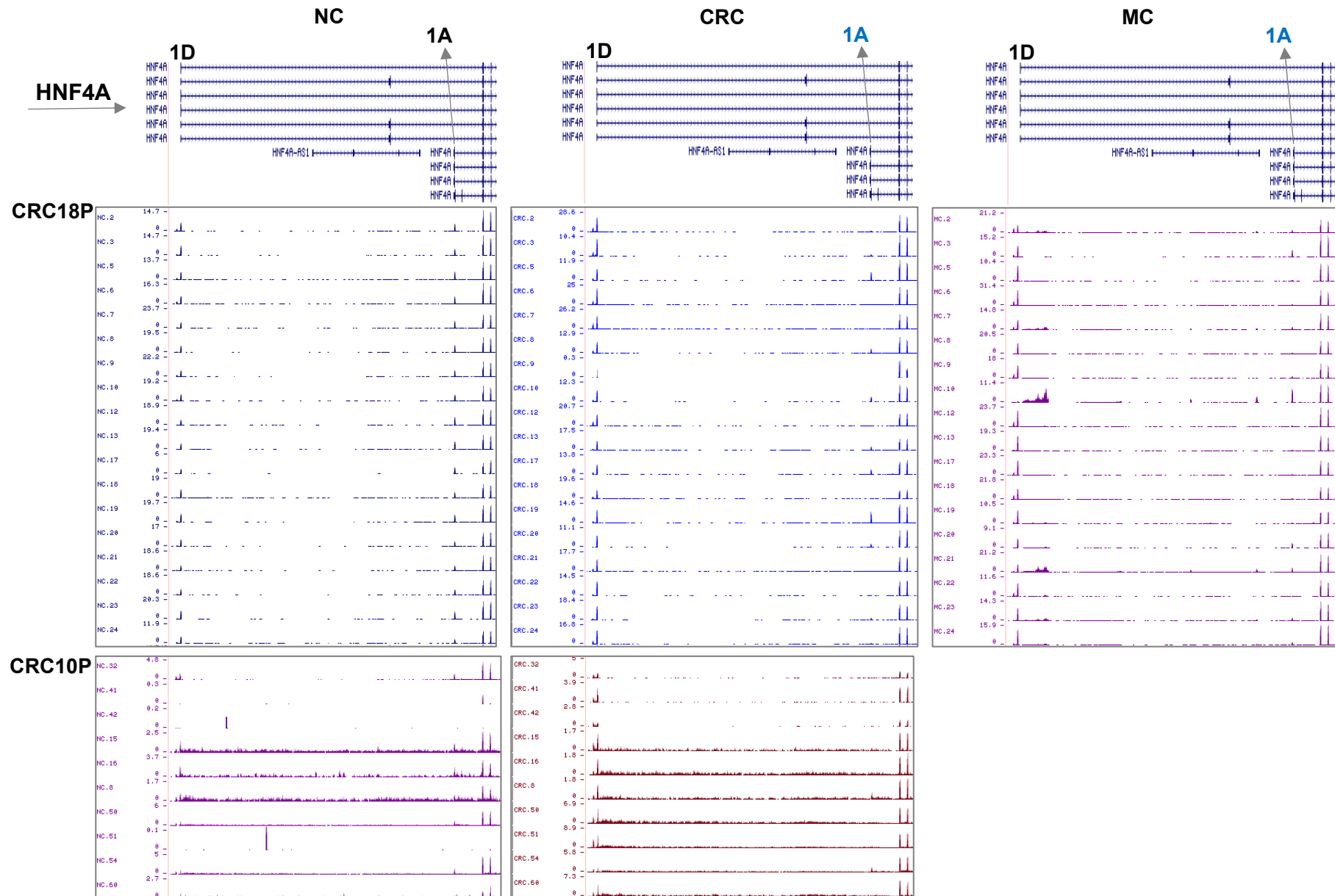

Supplement: Supplementary file 7 — Additional file 7: Figure S7. RNA-seq read coverage of HNF4A alternative first exons in CRC18P and CRC10P datasets. Exon 1A showed more exclusion/downregulation in CRC and MC samples (labeled in blue). [file 12876_2020_1288_MOESM7_ESM.pdf]

Figure S8.

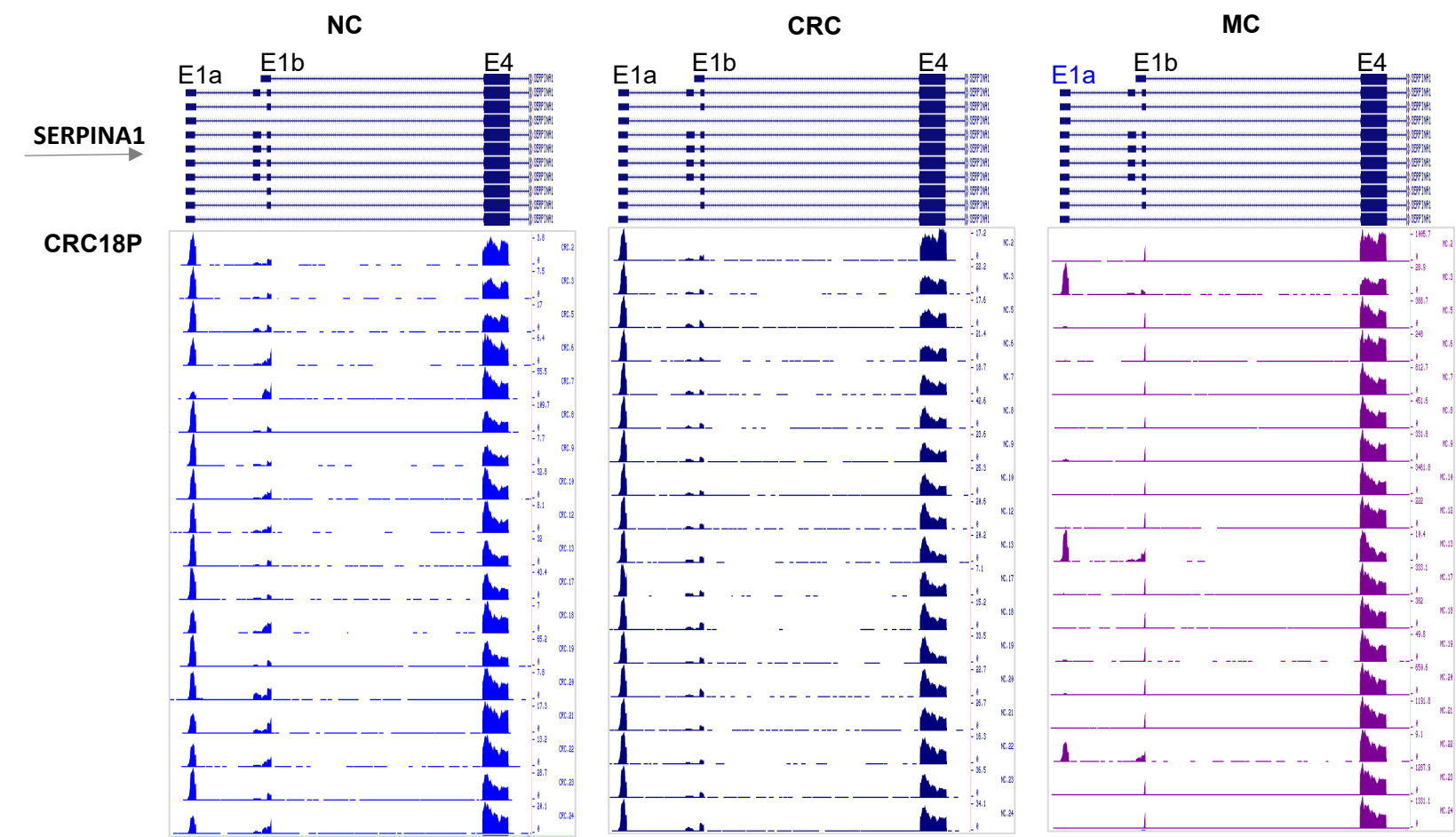

Supplement: Supplementary file 8 — Additional file 8: Figure S8. RNA-seq read coverage of SERPINA1 exon 1 to exon 4 in CRC18P dataset. Exon E1a showed more exclusion/downregulation in MC samples (labeled in blue). [file 12876_2020_1288_MOESM8_ESM.pdf]

Figure S9.

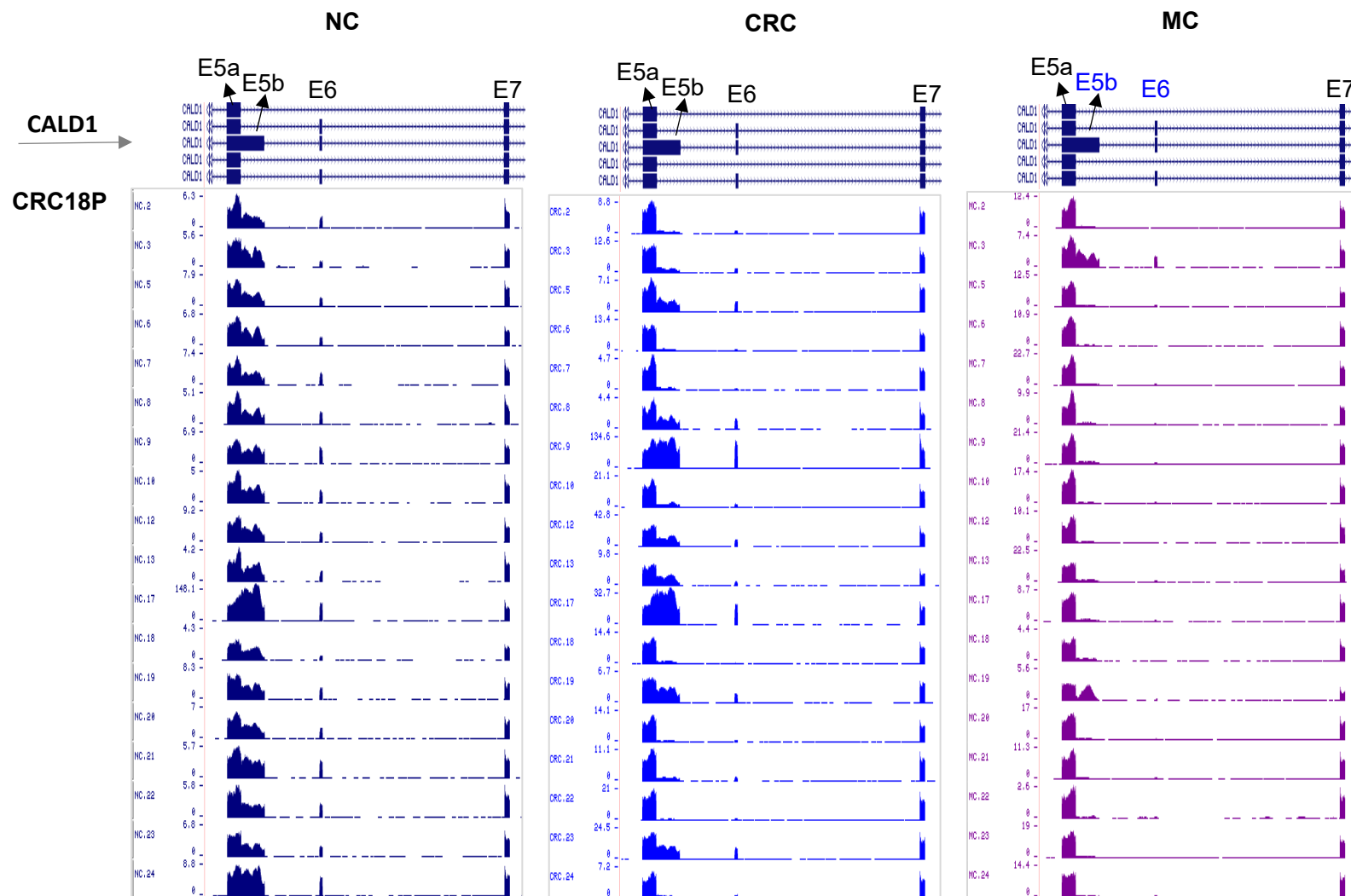

Supplement: Supplementary file 9 — Additional file 9: Figure S9. RNA-seq read coverage of CALD1 exon 5 to exon 7 in CRC18P dataset. Exons E5b and E6 showed more exclusion/downregulation in MC samples (labeled in blue). [file 12876_2020_1288_MOESM9_ESM.pdf]

**Figure S10.**

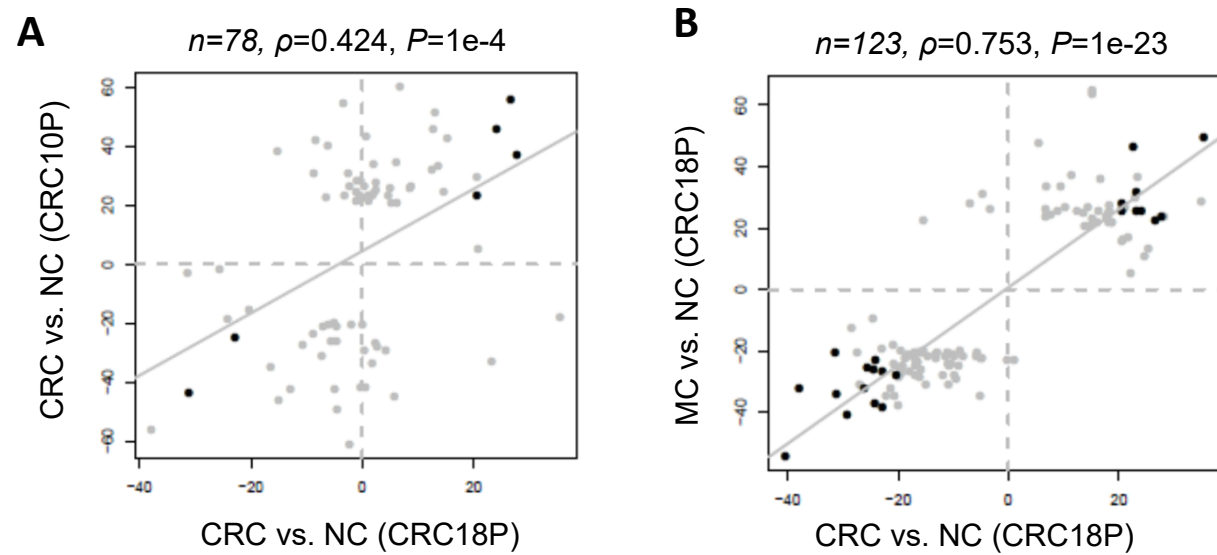

Supplement: Supplementary file 10 — Additional file 10: Figure S10. Scatter plot of ΔPSI_exon values. Each dot is an exon. Only exons with significant regulation (Wilcoxon rank-sum test P-value < 0.05 and |ΔPSI| > 20%) in either x or y axis were shown. Black dots represent events that are significant in both x and y axis. A linear regression line is based on all dots in the plot. The total number of dots (n), Spearman’s correlation coefficient value (ρ) and P-value (based on all dots) are shown. [file 12876_2020_1288_MOESM10_ESM.pdf]

Figure S11

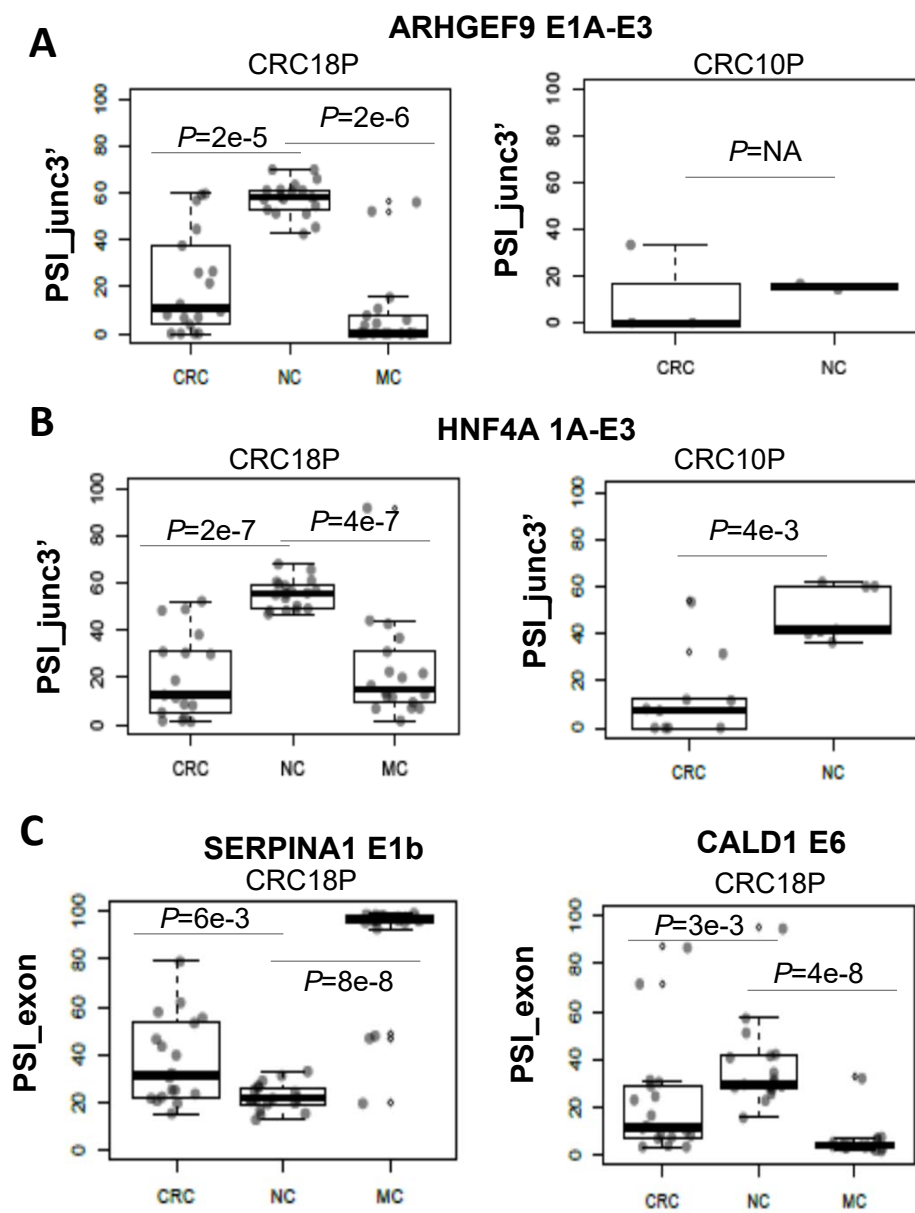

Supplement: Supplementary file 11 — Additional file 11: Figure S11. Boxplot of PSI values for genes in Figs. 4 and 5. Only AS events identified using PSI methods were plotted here. [file 12876_2020_1288_MOESM11_ESM.pdf]

Figure S12.

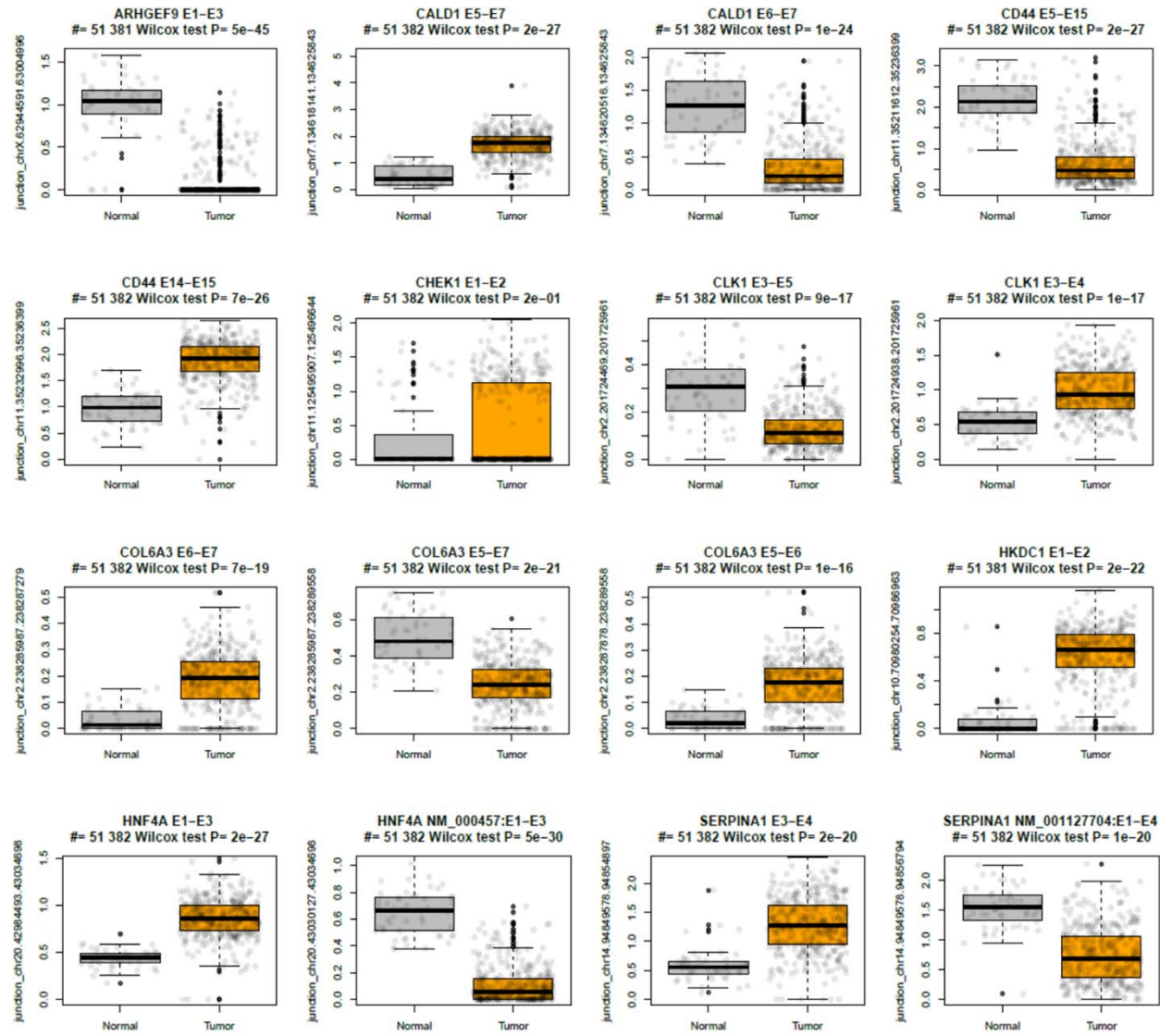

Supplement: Supplementary file 12 — Additional file 12: Figure S12. Splicing events identified in this study confirmed by TCGA junction expression data. Boxplots of junction usage of 16 junctions of 9 genes in 51 normal tissue and 382 CRC or metastatic tissue (tumor). P-values are based on Wilcoxon Rank-Sum Test. Nominal P-values are shown and adjusted P-values using Bonferroni correction can be found in the supplementary Table 7. Dots in the boxplot represent individual patient in TCGA. [file 12876_2020_1288_MOESM12_ESM.pdf]
