# Supplementary material for: Identification of novel alternative splicing isoform biomarkers and their association with overall survival in colorectal cancer
Source: BMC Gastroenterol. 2020 Jun 5;20:171. doi: 10.1186/s12876-020-01288-x (PMC7275609; doi:10.1186/s12876-020-01288-x)

Figure S13

A

		1			2			3		
	Spearman correlation	HKDC1	CALD1	CLK1	SERPINA1	CD44	COL6A3	ARHGEF9	HNF4A	
1	HKDC1	1.000	0.310	0.343	-0.184	-0.217	-0.073	-0.306	-0.111	junction_chr10.70980254.70986963
	CALD1	0.310	1.000	0.367	-0.149	-0.259	-0.235	-0.370	-0.245	junction_chr7.134618141.134625843
	CLK1	0.343	0.367	1.000	-0.054	0.003	-0.026	-0.307	-0.233	junction_chr2.201724938.201725961
2	SERPINA1	-0.184	-0.149	-0.054	1.000	0.421	0.418	0.376	0.286	junction_chr14.94849578.94856794
	CD44	-0.217	-0.259	0.003	0.421	1.000	0.529	0.444	0.267	junction_chr11.35211612.35236399
	COL6A3	-0.073	-0.235	-0.026	0.418	0.529	1.000	0.360	0.299	junction_chr2.238285987.238289558
3	ARHGEF9	-0.306	-0.370	-0.307	0.376	0.444	0.360	1.000	0.410	junction_chrX.62944591.63004996
	HNF4A	-0.111	-0.245	-0.233	0.286	0.267	0.299	0.410	1.000	junction_chr20.43030127.43034698

B

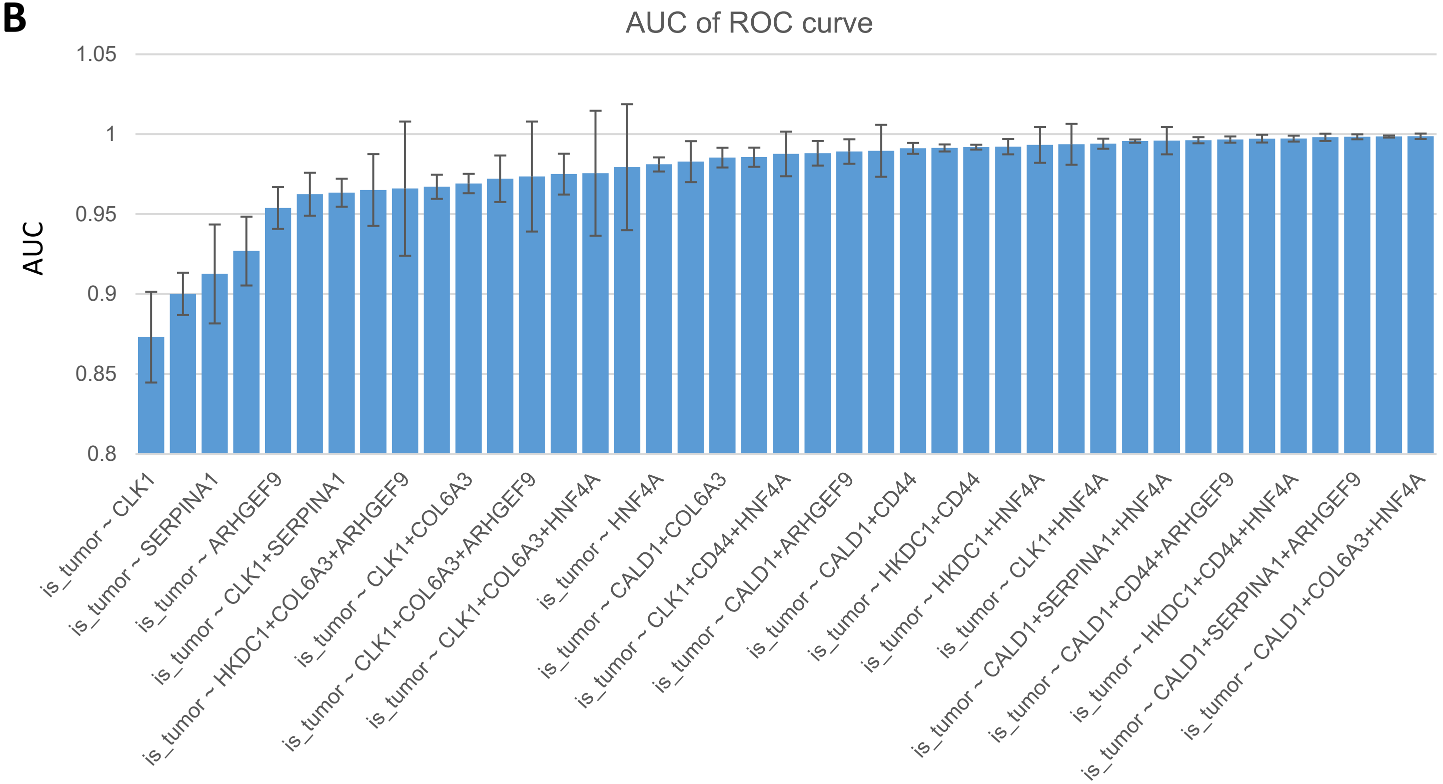

Supplement: Supplementary file 13 — Additional file 13: Figure S13. Logistic regression predicts sample type using junction usage data. (A) Spearman’s correlation coefficient of the junction usages of the TCGA data for the 8 genes. The 8 genes were separated to 3 groups (as indicated) based on the correlation values. (B) AUC scores of the ROC curves for the logistic regression using different number and combinations of the predictors. Average value of the five AUC scores for each formula were shown and the error bar is the standard deviation. Each AUC score was generated using randomly selected half of the data to train a logistic regression model and tested on the rest of the data. [file 12876_2020_1288_MOESM13_ESM.pdf]
